# Unofficial Editorial Notice

**Published:** 1845-03

**Authors:** Dan. Drake


### THE WESTERN JOURNAL.

New Series.—Vol. IV.—No. III.

LOUISVILLE, MARCH 1, 1845.

### UNOFFICIAL EDITORIAL NOTICE.

The undersigned takes this method of making known to those who may wish to consult him professionally, and also those who may have it in their power to send him facts on the diseases of the Valley of the Mississippi, fitted for the work in which he is engaged, that he will, after the present month, be in Cincinnati, throughout the approaching vacation of the Institute, except in the months of July and August.

DAN. DRAKE, M.D.

